# Intravoxel incoherent motion diffusion-weighted MR imaging in assessing and characterizing solitary pulmonary lesions

**DOI:** 10.1038/srep43257

**Published:** 2017-02-22

**Authors:** Qi Wan, Ying-Shi Deng, Jia-Xuan Zhou, Yu-Dong Yu, Ying-Ying Bao, Qiang Lei, Hou-Jin Chen, Ya-Hui Peng, Ying-Jie Mei, Qing-Si Zeng, Xin-Chun Li

**Affiliations:** 1 Department of Radiology, The First Affiliated Hospital of Guangzhou Medical University, No. 151 Yanjiangxi Road, Guangzhou, Guangdong 510120, China; 2 School of Electronic and Information Engineering, Beijing Jiaotong University, Beijing 100044, China; 3 Philips Healthcare, Guangzhou, Guangdong 510055, China

## Abstract

This study aimed to investigate the potential of intravoxel incoherent motion (IVIM) diffusion-weighted MR imaging in assessing solitary pulmonary lesions (SPLs). Sixty-two patients with pathologically confirmed SPLs, including 51 and 11 cases of malignant and benign lesions, respectively, were assessed. Diffusion weighted imaging (DWI) with 13 b values was used to derive apparent diffusion coefficient (ADC) and IVIM parameters, including true diffusion coefficient (D), pseudo-diffusion coefficient (D*), and perfusion fraction (f). Our results showed that, there was an excellent inter-observer agreement on the measurements of D and ADC between observers (inter-class correlation coefficient, ICC = 0.902 and 0.884, respectively). Meanwhile, f and D* showed good and substantial reproducibility (ICC = 0.787 and 0.623, respectively). D and ADC of malignant lesions were significantly lower than those of benign lesions (both P ≤ 0.001), while similar values were obtained in both groups for D* and f (both P > 0.05). In receiver operating characteristic (ROC) analysis, *D* showed the highest area under curve (AUC) for distinguishing malignant from benign lesions, followed by ADC. Accompanying signs of SPLs have specific features on IVIM maps. In conclusion, IVIM provides functional information in characterizing SPLs which is helpful to differential diagnosis. D and ADC have a significantly higher diagnostic value than f and D*.

Solitary pulmonary lesions (SPLs) are common entities encountered in chest imaging. Multi-slice Spiral Computed Tomography (MSCT) is the most common non-invasive method used to assess pulmonary lesions; however, some signs of lung cancer and benign lesions are similar, making it difficult to differentiate the two ailments based merely on morphologic features on CT images. Positron emission tomography (PET) using 18 F-fluorodeoxyglucose (FDG) and CT combination is more sensitive and specific in distinguishing lung cancer from benign lesions than CT; however, it also shows false-negatives in well-differentiated adenocarcinomas^1^ and false-positives in inflammatory nodules^2^.

The clinical application of pulmonary magnetic resonance imaging (MRI) was limited due to physical motion artifacts and technical limitations. However, with the development of technology in recent years, MRI has become a clinically feasible method for specific pulmonary problems. Diffusion weighted imaging (DWI) is sensitive to the molecular diffusion of water in biological tissues, and indexes such as cellularity, perfusion, tissue disorganization, extracellular space and other variables, can be exploited to diagnose a wide range of diseases^3^,^4^,^5^. Recently, DWI was shown to be a valuable tool for the detection and characterization of lung cancers and mediastinal lymph node metastases, comparable to or better than PET^6^,^7^,^8^. A few studies demonstrated that DWI along with apparent diffusion coefficient (ADC) measurement is helpful in discriminating malignant lesions from benign pulmonary nodules^9^,^10^. However, the ADC calculated by using the mono-exponential decay of signals ignores the effect of perfusion fraction in tissues, and does not represent true water diffusion. Intravoxel incoherent motion diffusion-weighted MR imaging (IVIM-DWI) could measure both true molecular diffusion in biological tissues and perfusion in the capillary network, which has the potential to better assess SPLs. IVIM-derived parameters include the true diffusion coefficient (D) and perfusion-related coefficient (pseudo-diffusion coefficient [D*] and perfusion fraction [f]). To our knowledge, few studies have focused on comparing IVIM-derived parameters and ADC for distinguishing malignant and benign SPLs^11^,^12^,^13^. IVIM-derived parameters have rarely been interpreted in detail in lung, including their reproducibility and influential factors. Furthermore, the appearance of accompanying signs of SPLs on IVIM maps, such as pleural retraction and pleural effusion, has not yet been reported. Such information is important in facilitating the IVIM application in lung and helps to understand the mechanism and value of IVIM parameters.

Therefore, in this study, we examined the diagnostic performances of ADC and IVIM parameters in differentiating SPLs. The sensitivity, specificity, and cut-off value of each diffusion parameter was calculated. The reproducibility of all parameters assessed by two radiologists was evaluated. Accompanying signs of SPLs on IVIM maps, such as pleural retraction and pleural effusion, have also been investigated.

## Results

### Pathological Findings

Pathological assessment revealed 51 and 11 cases of malignant tumors and benign lesions, respectively, among the 62 SPLs. The malignancy group included 28 adenocarcinoma, 13 squamous carcinoma, 1 small cell carcinoma, 2 large cell neuroendocrine carcinoma, 2 pleomorphic carcinoma, 1 lymphoepithelioma-like carcinoma, 1 undifferentiated type of non-small cell lung cancer, 2 lymphatic epithelioma, and 1 malignant inflammatory myofibroblastic tumor cases. The benign tumor group included 1 pulmonary cryptococcosis, 2 tuberculosis, 1 lung abscess, 3 inflammatory granuloma, 1 sclerosing pneumocytoma, 2 hamartoma and 1 benign spindle cell tumor cases.

### MRI characteristics

**Figure 1:**
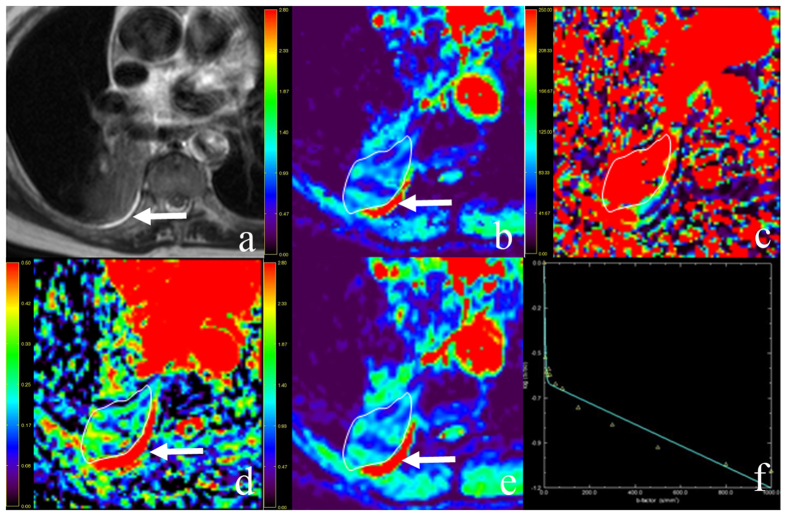
Representative imaging characteristics in a 65-year-old man with right lower lobe adenocarcinoma. (**a**) T2-weighted image. (**b**) The D map. (**c**) The D* map. (**d**) The f map. (**e**) Apparent diffusion coefficient (ADC) map. (**f**) The bi-exponential fitting of the diffusion signal decay. The tumor demonstrates slightly high signal on T2WI and mixed color on D and ADC maps. Dispersed blue and violet areas (representing regional restricted diffusion) were observed, which indicates a malignant tumor. The f map demonstrates a relatively high perfusion fraction region compared to the lung. The D* map shows a high pseudo-diffusion values in the tumor tissue. Pleural effusion (arrow) demonstrates as hyperintensity on T2WI as well as high value on D, ADC and f maps (encoded in a red color).

**Figure 2:**
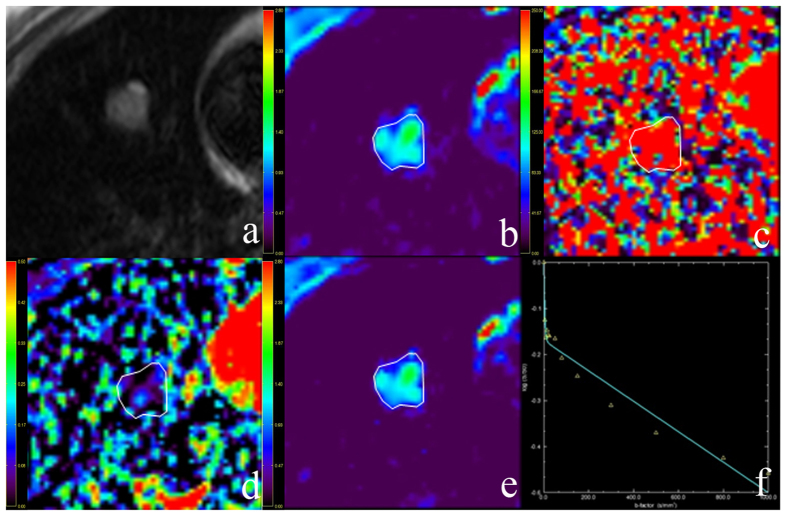
Representative imaging characteristics in a 38-year-old woman with right middle lobe benign sclerosing pneumocytoma. (**a**) T2-weighted image. (**b**) The D map. (**c**) The D* map. (**d**) The f map. (**e**) Apparent diffusion coefficient (ADC) map. (**f**) The bi-exponential fitting of the diffusion signal decay. The tumor shows slight increase on T2 signal intensity and slightly high signal on D and ADC maps (encoded in cyan and green colors), which indicates a benign tumor. The f map demonstrates a relatively low perfusion fraction region compared to the lung. The D* map shows a high pseudo-diffusion values in the tumor tissue.

**Figure 3:**
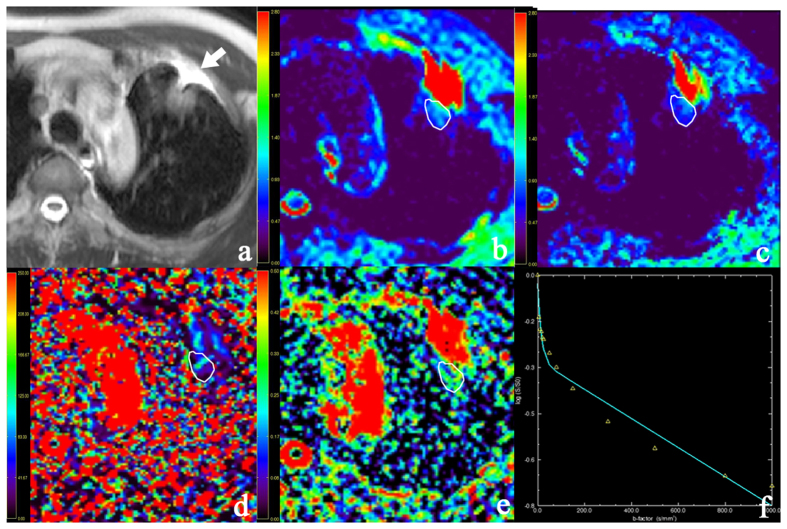
Pleural retraction in a 52-year-old man with left upper lobe adenocarcinoma. (**a**) T2-weighted image. (**b**) Apparent diffusion coefficient (ADC) map. (**c**) The D map (**d**) The D* map. (**e**) The f map. (**f**) The bi-exponential fitting of the diffusion signal decay. Pleural retraction (arrow) demonstrates triangle-like hyper-intensity on T2WI. It possesses high ADC, D, f values and low D* values. The tumor (partly showed) show impeded diffusion on ADC and D maps (encoded in blue color).

The longest lesion diameters were in the range of 1.0–9.7 cm, with a mean of 3.2 cm. Figures 1 and 2 show representative imaging characteristics of two cases pathologically confirmed as benign tumor and lung cancer, respectively. Figures 1 and 3 show characteristics of pleural effusion and pleural retraction on IVIM maps.

### Mono-exponential and bi-exponential analysis of diffusion data

**Figure 4:**
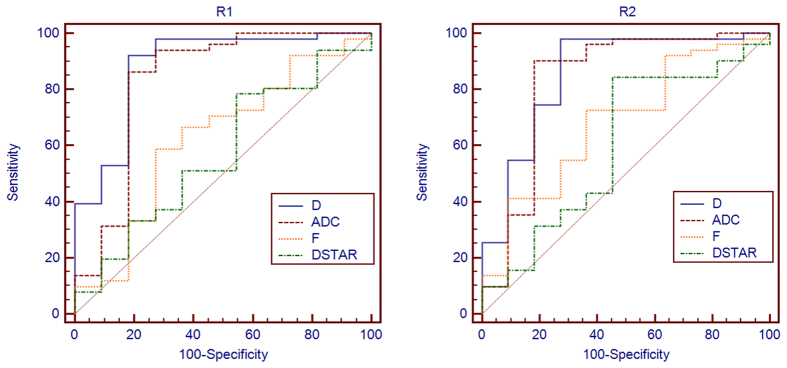
Receiver operating characteristic (ROC) curves of IVIM and ADC obtained by two radiologists (R1 and R2) used for differentiating benign from malignant pulmonary lesions. Area under the curve (AUC) for all parameters were obtained by observers 1 (R1) and 2 (R2). AUC value for D was slightly higher than that of ADC (R1: 0.884 vs. 0.832, P = 0.491; R2: 0.856 vs. 0.831 P = 0.645).

ADC values were significantly lower in malignant tumors than in benign lesions (Radiologist 1: 1.15 ± 0.25 × 10^−3^ mm^2^/s vs. 1.82 ± 0.62 × 10^−3^ mm^2^/s, P = 0.001; Radiologist 2: 1.15 ± 0.22 × 10^−3^ mm^2^/s vs. 1.61 ± 0.48 × 10^−3^ mm^2^/s, P = 0.001). As for IVIM parameters, the D values obtained for malignant lesions were significantly lower compared with those of benign lesions (Radiologist 1: 0.94 ± 0.21 × 10^−3^ mm^2^/s vs. 1.41 ± 0.32 × 10^−3^ mm^2^/s; P < 0.001; Radiologist 2: 0.92 ± 0.2 × 10^−3^ mm^2^/s vs. 1.38 ± 0.35 × 10^−3^ mm^2^/s; P < 0.001). No significant differences were obtained in f and D* between the lung cancer and benign lesion groups (P > 0.05). The ADC values and IVIM parameters for both groups are summarized in Table 1 and Fig. 4.

### Diagnostic performances of MRI parameters

*D* showed the highest area under curve (AUC) for distinguishing malignant from benign lesions, with a value of 0. 884 (95% Confidence Interval [CI] 0.777–0.951) obtained by Radiologist 1; it was followed by ADC, with AUC = 0.832 (95%CI, 0.716–0.915). The optimal thresholds for differentiating malignant from benign lesions were 1.20 × 10^−3^ mm^2^/s for D, which yielded sensitivity and specificity of 92.16% and 81.82%, respectively, and 1.32 × 10^−3^ mm^2^/s for ADC, with sensitivity and specificity of 86.27% and 81.82%, respectively. Both f and D* were poor malignancy markers with AUC values of 0.624 (95%CI 0.492–0.744) and 0.57 (95%CI 0.419–0.673), respectively.

**Figure 5:**
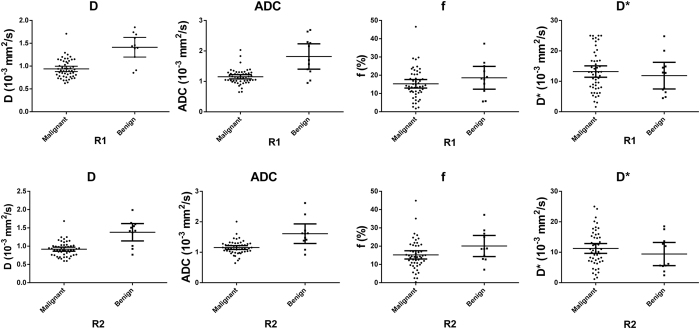
ADC and IVIM parameters (mean ± 95%CI) measured by two radiologists for lung lesions. Parameters obtained by radiologists 1 and 2 are represented by R1 and R2, respectively. D and ADC showed a significant difference (P < 0.001, P = 0.001) between malignant and benign lesions. There was no difference in f and D* between two groups.

Meanwhile, the AUC values obtained by Radiologist 2 were 0.856 and 0.831 for D and ADC, with 1.25 × 10^−3^ mm^2^/s and 1.37 × 10^−3^ mm^2^/s as cutoff values, respectively. Sensitivity and specificity of these cutoff values were 98.04% and 72.73%, respectively, for D, and 90.2 and 81.82%, respectively, for ADC. The results of ROC-based analyses are shown in Table 2. ROC curves for ADC, D, D*, and f values are illustrated in Fig. 5.

### Inter-observer reproducibility

**Figure 6:**
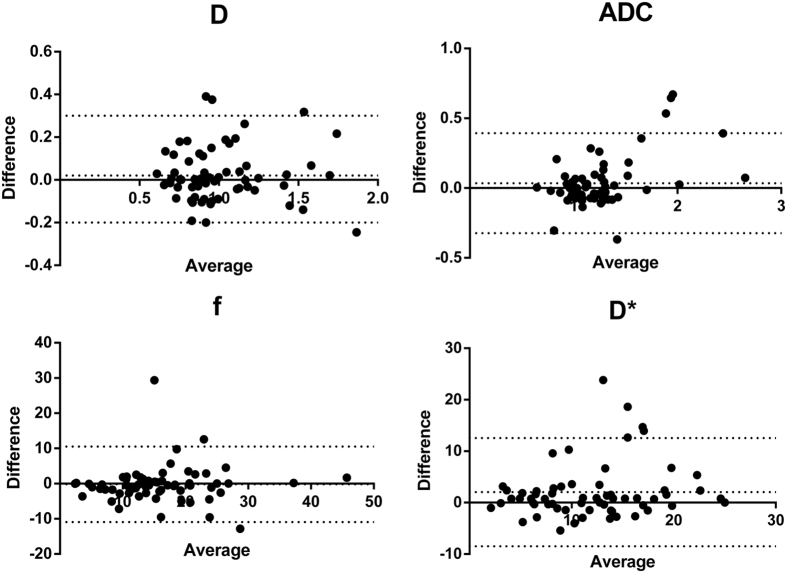
Bland–Altman analysis of differences between repeated measurements by both radiologists.

Inter-observer agreement between the two radiologists was great for D (ICC = 0.902) and ADC (ICC = 0.884), followed by f (ICC = 0.787). However, ICC was relatively lower for D* (0.623). Bland–Altman analyses for inter-observer measurements are shown in Fig. 6.

## Discussion

IVIM is a technique with the potential of simultaneously assessing both tissue perfusion and diffusion by using a single sequence. The diffusion coefficient (D) and perfusion related coefficients (D* and f) were assessed separately, thus allowing more accurate lesion assessments. The present study clarified the perfusion and diffusion features of solitary pulmonary nodules based on the IVIM model, using multiple b values. However, the number of b value used in IVIM does not form a unified standard, varying from 4 to more than 10 according to the literature. However, due to the relatively low stability and reproducibility of data obtained with low b values^14^, more b values are recommended <200 s/mm^2^. The number of b values <50 s/mm^2^ should be at least 2, to assess the perfusion fraction more accurately^15^. In this study, 13 b values were adopted, with 5 lower than 50 s/mm^2^ and 8 lower than 200 s/mm^2^, to ensure accuracy and reproducibility of IVIM-derived parameters.

This study demonstrated that D values for lung cancer were significantly lower than those of benign nodules, corroborating a previous study^13^. This change can be interpreted as increased cellular proliferation and decreased extracellular space in lung cancer compared with benign pulmonary lesions. Furthermore, the current study found that D showed diagnostic properties greater than those of ADC in differentiating lung cancer from benign lesions; D could reduce false negative rate in identifying malignant lesions compared with ADC. This may be explained by the fact that IVIM removal of perfusion contamination could be beneficial for revealing the increased cellular density in malignancies. However, the difference of diagnostic performance between D and ADC was not statically significant. This may be due to the fact that the perfusion portions of malignant and benign groups were nondiscriminatory (as mentioned below), the difference of ADC between both groups mainly ascribed to the diffusion portion. Thus, similar diagnostic performances were obtained for D and ADC.

Despite a better performance, there remains an overlap in D and ADC values between benign and malignant lesions; this may be associated with several factors. First, lung cancer is pathologically diverse. Lung adenocarcinoma had relatively higher D and ADC values compared with other lung cancer subtypes^10^. Hereinto, some mucous adenocarcinomas possess even higher ADC and D values that can exceed the threshold values (approximately 1.20 × 10^−3^ mm^2^/s for D and 1.32 × 10^−3^ mm^2^/s for ADC) and is difficult to be distinguished from benign lesions. In this study, a mucous adenocarcinoma reach a value of 1.71 × 10^−3^ mm^2^/s for D and 2.03 × 10^−3^ mm^2^/s for ADC. This may be related to its abundant mucus component. Second, due to conditions such as inflammatory cell infiltration, purulent exudate, fibrous tissue hyperplasia, and increased cell viscosity, some infectious diseases show impeded diffusion on DWI, making their ADC or D values decrease and overlap with those of malignant lesions.

In this study, both perfusion-related diffusion coefficients, D* and f, were not significantly different between malignant and benign lesions. According to the IVIM theory, D* is related to the average blood flow rate and mean blood capillary length^16^. In this study, D* values of benign and malignant lesions showed no statistically significant differences, in agreement with previous studies^11^,^13^. This may be associated with poor reproducibility and the physiological anatomy of the lung. The arteriovenous network widely exists in the lung parenchyma and fast blood flow may cause a relatively high D* of the whole lung parenchyma. In addition, D* is sensitive to macroscopic movements; therefore, data collection during free breathing might also increase D* noise in the lesions. Furthermore, D* was shown to have a relatively lower reproducibility in this study. These might resulted in a poor potential for D* in differentiating malignant from benign SPNs.

f is the fractional volume of capillary blood flowing^16^. Lung parenchyma containing air appeared as low intensity signals on the f map; this resulted in a good contrast between lung parenchyma and lesions; meanwhile, the pulmonary vasculature remained visible on the f map. It is worth noting that pleural retraction and pleural effusion are characterized by high signal intensity on the f map (i.e., with high f values) and low intensity signals on D* map. This suggests that f is more exactly the ratio of the volume of MRI-visible water flowing in each voxel, while D* seems to be more objective to reflect the blood supply of pulmonary lesions. Deng *et al*.^11^ found that the f value of pulmonary inflammatory granuloma is significantly higher than that of lung cancer. However, in our study, no differences were found between benign and malignant lesions, corroborating Yuan *et al*.^13^ This, on one hand, may be due to differences in b-values and patient populations. Indeed, benign cases with rich blood supply, such as infectious granulomas and pulmonary sclerosing hemangiomas, were included in this study, alongside a small number of lung cancer cases not rich in blood supply; this might explain the blood supply overlap between benign and malignant lesions. On the other hand, f not only reflects perfusion properties, but is also affected by other factors, such as relaxation effects and T2 contribution^17^. This explains why lung parenchyma containing air with a low T2 value also possesses low f, while pleural effusion with a high T2 value equally has high f. Carinci *et al*.^18^ used single shot turbo spin-echo sequence with stimulated-echo preparation and electro-cardiograph synchronization to quantify blood volume fraction of the human lung. In their study, short TE was adopted, reducing T2 signal attenuation, to more accurately evaluate blood flow in the lung parenchyma; the f values reported in their study were in good agreement with previous reports using contrast-enhanced magnetic resonance angiography. Therefore, f value may be a potential index in evaluating blood supply of pulmonary lesions, but its stability and application value need further research.

This study has some limitations. First, in order to guarantee accuracy, all cases in this study were confirmed by pathology, which resulted in a relatively small patient population with benign lesions. Second, the regions of interest were selected in solid parts instead of the entire lesions, which might lead to selection bias owing to the histologic heterogeneity of tumors. In addition, the retrospective nature of this study may also be considered a limitation. Further prospective studies with a larger sample size will be needed to compare the diagnostic efficiency of different DWI models in differentiating benign and malignant pulmonary lesions of various subtypes.

In conclusion, our findings suggest that IVIM parameters derived from the bi-exponential fitting provide more information in characterizing SPLs, compared with ADC values from the mono-exponential fitting model. D and ADC have a significantly higher diagnostic value than f and D*.

## Methods

### Patients

This retrospective study was approved by The Institutional Review Board of The First Affiliated Hospital of Guangzhou Medical University, and informed consents were obtained from all patients. This study was conducted in accordance with the Declaration of Helsinki. Between July 2014 and February 2015, 84 consecutive patients with suspected solitary pulmonary nodules or masses underwent routine MRI and IVIM-DWI of the lung. Of these, 22 subjects were excluded because of one or more of the following: 1, Operations or biopsies were not performed (n = 7); 2, inadequate confirmation of pathological findings (n = 3); 3. Masses or nodules had a short axis diameter under 1 cm or with an air-containing area larger than 1/3 of diameter (n = 8); 4. Unsatisfactory imaging quality (n = 4). The final study population contained 62 patients (22 women, 40 men; mean age, 56 years; age range, 30–81 years) with pathologically diagnosed SPLs.

### MRI scanning

All patients were examined with a 3.0-T MRI (Achieva, Philips Healthcare, Best, The Netherlands), using a body phase array coil. The MRI sequences including: axial gradient echo T1-weighted (T1W) imaging; axial and coronal turbo spin-echo T2-weighted (T2W) imaging. Routine MR images were obtained during end-inspiration breath-holding. The following parameters were used for T2WI: T2WI/TSE, TR/TE = 998/80 ms, NSA = 1, FOV = 340 mm × 430 mm, matrix 640 × 640, thickness/gap = 5.0 mm/0.5 mm, scan time 24 s. Coronal T2WI: T2WI/TSE, TR/TE = 1131/80 ms, NSA = 2, FOV = 430 mm × 430 mm, matrix 432 × 432, thickness/gap = 5.0  mm/0.5 mm, scan time 27 s.

Multiple b value diffusion-weighted imaging scan was acquired using a single-shot echo-planar imaging pulse sequence with free breathing. Parallel imaging was used, and fat was suppressed using spectral presaturation inversion recovery (SPIR). The parameters were as follows: multiple b value = 0, 5, 10, 15, 20, 25, 50, 80, 150, 300, 500, 800, and 1000 s/mm^2^; repetition time, 1111 ms; echo time, 55 ms; field of view, 375 mm; matrix, 256 × 256; section thickness, 3.0 mm with 0.3 mm gap. scan time 7 mins 31 s.

### MRI analyses

DW data were post-processed with mono- and bi-exponential IVIM models. ADC values for lung lesions were derived from 13 b values calculated using a mono-exponential fit of signal intensity with the equation (1):


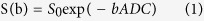


For the bi-exponential IVIM model, the relationship between DWI signal intensity and the b factors can be expressed as equation (2):


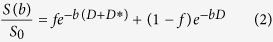


where S (b) is the signal intensity at different diffusion values, S_0_ the signal intensity at b = 0 s/mm^2^, f the fraction of perfusion, D the diffusion parameter representing pure molecular diffusion, and D* the pseudo-diffusion parameter representing incoherent microcirculation within the voxel. Data was fitted with the Levenberg-Marquardt nonlinear least squares algorithm.

The original DWI images obtained were transferred to a workstation and post-processed with PRIDE software (Phillips Medical Systems, Best, The Netherlands). Mono- and bi- exponential models were used to calculate the corresponding ADC maps and D, and f and D* pseudo color pictures based on calculated values of each parameter. ROI (region of interest, ROI) was manually drawn on the solid part of the lesion at the level of maximum transverse diameter, avoiding liquefaction, necrosis and hemorrhage by two experienced radiologists (R1:Q.W. and R2:X.C.L. with 5 years and 15 years of the magnetic resonance diagnostic experience respectively) blinded to the pathologic reports.

### Statistical analyses

Data is presented as mean ± standard deviation (SD). IVIM parameters (D, f, and D*) and ADC between malignant and benign groups were compared by Mann-Whitney U test. Receiver operating characteristic (ROC) curve analysis was performed to evaluate the diagnostic performances of IVIM parameters and ADC in predicting malignancy, and determine suitable threshold values. The areas under the ROC curves (AUCs) for IVIM parameters were compared to those of ADC; the optimal cutoff value was defined as the point yielding the best sensitivity and specificity for the differentiation. The inter-observer reproducibility, i.e. the difference between measurements made by the two observers, was assessed using inter-class correlation coefficient (ICC) and Bland–Altman plot. Statistical analyses were performed with SPSS (version 19.0, IBM Corporation, America) and MedCalc software (version 15.2.2). The tests were two-tailed, and a value of P < 0.05 was considered statistically significant. The ICC values were considered to indicate excellent agreement if they were greater than 0.85, good agreement if they were in the range of 0.75–0.84, and substantial agreement if they were in the range of 0.60–0.74.

## Additional Information

**How to cite this article**: Wan, Q. *et al*. Intravoxel incoherent motion diffusion-weighted MR imaging in assessing and characterizing solitary pulmonary lesions. *Sci. Rep.* **7**, 43257; doi: 10.1038/srep43257 (2017).

**Publisher's note:** Springer Nature remains neutral with regard to jurisdictional claims in published maps and institutional affiliations.

## Figures and Tables

**Table 1:** IVIM parameters and ADC of malignant and benign SPLs.

	Observer	Malignant (n = 51)	Benign (n = 11)	P	ICC
ADC	R1	1.15 ± 0.25	1.82 ± 0.62	0.001	0.884
	R2	1.21 ± 0.30	1.84 ± 0.54	0.001	
D	R1	0.94 ± 0.21	1.41 ± 0.32	<0.001	0.902
	R2	0.92 ± 0.20	1.38 ± 0.35	<0.001	
f	R1	15.30 ± 8.27	18.61 ± 9.31	0.2	0.787
	R2	15.22 ± 8.05	20.09 ± 8.61	0.061	
D*	R1	13.21 ± 6.66	11.87 ± 6.51	0.467	0.623
	R2	11.26 ± 5.73	9.42 ± 5.66	0.298	

SPL: Solitary pulmonary lesions, R1: Radiologist 1, R2: Radiologist 2, ICC: Inter-class correlation coefficient, ADC: apparent diffusion coefficient, D: diffusion coefficients, f: perfusion fraction, D*: pseudo-diffusion coefficient.

**Table 2:** Diagnostic Performance of D and ADC for differentiation of malignancies from benign lesions.

	Observer	AUC	95% CI	Cut-off	Sensitivity (%)	Specificity (%)	+LR	−LR
ADC	R1	0.832	0.716–0.915	1.32	86.27	81.82	4.75	0.17
	R2	0.831	0.714–0.914	1.37	90.20	81.82	4.96	0.12
D	R1	0.884	0.777–0.951	1.20	92.16	81.82	5.07	0.096
	R2	0.856	0.743–0.932	1.25	98.04	72.73	3.59	0.027

R1: Radiologist 1, R2: Radiologist 2, AUC: Area under ROC curve. ADC: apparent diffusion coefficient, 95% CI: 95% confidence intervals, D: diffusion coefficients, +LR: positive likelihood ratio, −LR: negative likelihood ratio.
